# Active temperature-humidity management seating for athletes in waiting areas

**DOI:** 10.1016/j.isci.2026.114984

**Published:** 2026-02-11

**Authors:** Chong-ran Zhao, Feng-shang Wang, Chao-ran Liu, Lin Zhang, Liang Chu

**Affiliations:** 1 Shenyang Sport University, Shenyang, Liaoning 110000, China; 2 School of Electronics and Information, Hangzhou Dianzi University, Hangzhou 310018, China

**Keywords:** health technology, temperature control in health technology, applied sciences

## Abstract

The competitive achievement and recovery of athletes during pre-competition waiting periods is significantly influenced by their cutaneous thermal-humidity microenvironment. Existing environmental systems in athletic venues have issues like high maintenance cost and lack of individualized regulation. This study developed an intelligent adaptive seating system with multifunctional climate control, incorporating AHT20 temperature and humidity sensor. It monitored real-time temperature and relative humidity data on facial surfaces of athletes to form a physiological profile of their post-exercise thermo-humid state. Through intelligent analysis, the system can automatically tune the built-in heating, cooling, and dehumidifying devices to optimize the seat’s microenvironment. Preliminary results indicate that the system effectively alleviates high thermal-hygrometric states, promotes heat dissipation, and creates conditions conducive to respiratory comfort. These findings suggest the system offers a feasible intelligent solution that may potentially support athlete recovery.

## Highlights

• An adaptive smart seat was created for athletes in the pre-competition waiting time
• The system monitors facial temperature and humidity to form “physiological fingerprint”
• The smart seat can auto-tune heating/cooling/dehumidifying via intelligent data analysis
• Stable regulation of the microenvironment enhances the efficiency of athletes’ recovery

## Graphical abstract

**Figure:**
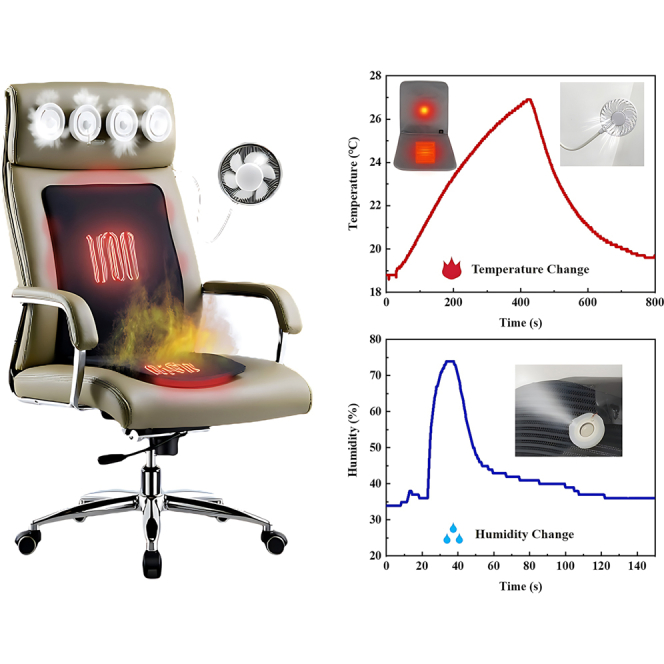

## Introduction

With the rapid development of the global sports industry, competitive sports have garnered significant international interest owing to their distinctive charm and challenges. Elite athletic disciplines are highly esteemed for their challenging technical skills and artistic expression, as exemplified by ice dance and figure skating. Nonetheless, these high-performance competitive events place stringent demands on athletes’ physiological state management. During pre-competition or training waiting periods, athletes must swiftly recover from intense physical exertion. The thermal and moisture comfort of their body surface directly influences their physical recovery, psychological state, and subsequent competitive performance. Inadequate management of post-exercise thermal load and excessive sweating can result in muscle stiffness, increased fatigue, and a heightened injury risk, severely limiting athletes’ ability to maintain high-level performance.^1^^,^^2^^,^^3^^,^^4^

Specific environmental conditions within many athletic venues present significant thermoregulatory challenges. For instance, high humidity and limited ventilation, common in indoor stadiums, can impede sweat evaporation. This may lead to excessive body heat accumulation and thermal discomfort, particularly during inactive periods. Conversely, the frigid environment of ice rinks, required for sports such as ice dance and figure skating, can cause rapid cooling of the body surface, which may compromise thermal insulation and potentially provoke respiratory discomfort.^5^^,^^6^^,^^7^^,^^8^ Elite athletic performance not only requires exceptional physical conditioning but also emphasizes the importance of psychological stability. Inadequate temperature and humidity conditions can directly disrupt athletes’ mental preparation, leading to increased tension or anxiety, and subsequently affecting their performance in sports.^9^^,^^10^ Therefore, the precise regulation of temperature and humidity is pivotal for maintaining peak performance in competitive sports.

Recently, two main categories of solutions have been implemented to offer athletes more comfortable waiting environments: large-scale intelligent environmental control systems and personal wearable technologies.^11^^,^^12^ For example, the National Aquatics Center has set up an intelligent environmental control system by integrating sensor networks, intelligent control platforms, and various environmental regulation devices, such as air conditioning and ventilation equipment. The system operates through a “perception-analysis-decision-execution-feedback” closed-loop mechanism, ensuring optimal comfort for athletes during waiting periods and thus improving their training and competitive performance.^13^^,^^14^^,^^15^^,^^16^ Nonetheless, these intelligent environmental control systems still face several limitations.^17^ Their complexity and high maintenance costs prevent widespread adoption, and more importantly, they lack the ability to regulate environments based on athletes’ dynamic and individualized physiological needs. On the other end of the spectrum, personal wearable technologies, such as cooling vests or smart textiles, offer high personalization but can be cumbersome, invasive, and interfere with an athlete’s mental and physical pre-competition preparation. Therefore, both approaches present significant drawbacks, leaving a gap for a solution that offers real-time, personalized physiological regulation in a non-invasive manner.

To tackle these challenges, we have designed a multifunctional adaptive intelligent control seat system to meet the unique waiting-environment needs of athletes, based on the research foundation of our group in motion monitoring.^18^^,^^19^ The core innovation of this system lies in its integration of the AHT20 temperature and humidity sensor. This provides real-time monitoring of the ambient conditions around athletes, supplying critical environmental data for comprehensive physiological monitoring. These physiological parameters are essential for evaluating thermal comfort and tracking recovery progress. Specifically, surface temperature serves as a direct indicator of an athlete’s body heat load and dissipation efficiency. Furthermore, facial local relative humidity (RH), particularly near respiratory zones, not only measures skin perspiration but also indirectly reflects respiratory gas humidity, offering critical physiological insights into respiratory health, fluid balance, and heat dissipation via respiration. By intelligently analyzing these physiological profile data, the system adaptively adjusts its built-in heating, cooling, and humidity regulation devices to create personalized microenvironments tailored to athletes’ needs. This design is to provide athletes with a comfortable and personalized waiting environment that supports the maintenance of optimal physiological and psychological states.

Our intelligent seating system aims to occupy a unique and advantaageous middle ground. As shown in Table 1, it maintains the highly personalized characteristics of wearable devices while adopting a completely non-intrusive approach. By embedding this technology into the seats in the waiting area, it can support the physical recovery of athletes without hindering them and without requiring complex renovations of the venue. This makes it an economical and athlete-centered solution that can enhance an athlete’s performance by optimizing the critical recovery phase before the competition.

**Table 1:** Comparison of thermal-humidity regulation technologies for athletes

Feature	Our Intelligent Adaptive Seating	Smart Venue Environmental Systems^20^^,^^21^	Wearable Cooling/Heating Garments^22^^,^^23^
Personalization level	high	low	high
Control feedback	direct and automated	indirect	variable
Portability and flexibility	high	low	very high
Cost-effectiveness	moderate	very high	moderate
Ease of use and maintenance	very high	high	moderate

### Design

#### Hardware driver design

**Figure 1:**
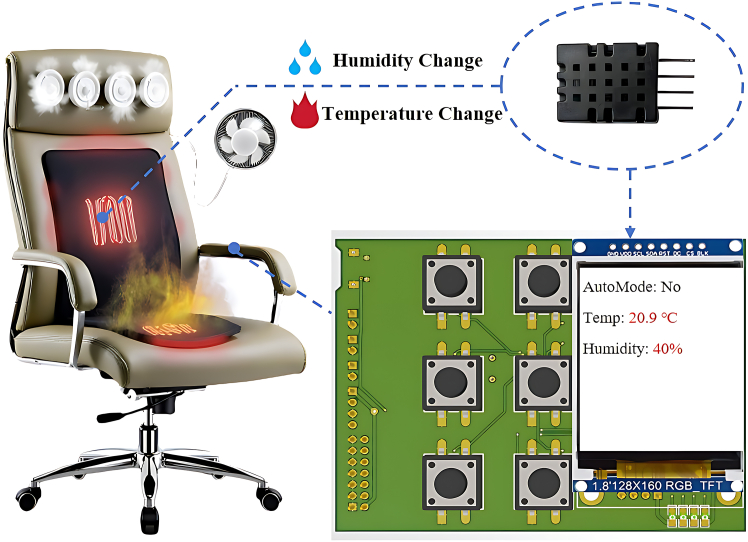
The schematic diagram of the intelligent seat integrated temperature and humidity sensor with main control circuit board

This study designed a multifunctional adaptive intelligent control seat system based on biosensors to meet the personalized physiological recovery and thermal comfort needs of performance-oriented ice sports athletes during waiting periods. Figure 1 illustrates the structural diagram of this intelligent control seat. The system can intelligently regulate the temperature and humidity of the waiting environment by integrating AHT20 temperature and humidity sensor, button control modules, and data display components. The system functions through the following workflow: first, biosensors continuously monitor the temperature and humidity data in the athlete’s surface microenvironment, especially in the facial region. These analog signals are converted into digital signals using a high-performance analog-to-digital converter (ADC) and sent to a microcontroller unit, such as the STM32. Simultaneously, the system includes a button control interface, enabling users to make real-time adjustments based on their individual physiological perceptions or preferences. After receiving physiological data from the sensors and user input signals, the microcontroller performs a systematic analysis using pre-programmed intelligent logic embedded in the software. The analysis results are then converted into precise control commands to manage the built-in heating, cooling, and humidity regulation actuators of the seat.^24^ The system displays the real-time operating mode and collected surface temperature-humidity data on a liquid crystal display (LCD) screen, providing athletes with immediate feedback.

During data processing and decision-making, the microcontroller compares the digital signals from the biosensors with preset physiological comfort thresholds. Based on this analysis, the system accurately controls the seat microenvironment actuators through various driving circuits. For example, if the system detects deviations in the athlete’s facial surface temperature or ambient humidity from the optimal physiological range, it automatically adjusts the operation of the heating, cooling, and humidity regulation devices to quickly maintain an ideal recovery environment. This adaptive control mechanism, which is driven by real-time physiological feedback, is essential for achieving intelligent and personalized temperature and humidity regulation.

Furthermore, to enhance system visualization and user interaction, the system employs the serial peripheral interface (SPI) protocol to drive the LCD screen. This display provides intuitive, real-time information on the current surface microenvironment’s temperature, humidity, and system operating mode, greatly enhancing data readability and usability. This intuitive physiological feedback not only aids athletes in self-monitoring but also allows users to make personalized adjustments to the seat’s temperature and humidity according to their physiological perceptions or recovery needs, thus achieving collaborative optimization of the physiological environment between humans and machines.

The sensor, as the front-end component for signal acquisition, plays a crucial role in the effective operation of the entire system. The AHT20 temperature and humidity sensor is utilized to collect environmental data in this system. A signal processing module allows the AHT20 sensor to maintain a stable operating state, transmitting its data to the embedded microcontroller system via the inter-integrated circuit (I^2^C) protocol for further processing. The AHT20 sensor offers RH measurement accuracy of ±2% and temperature measurement accuracy of ±0.3°C at 25°C.^25^^,^^26^ Utilizing the AHT20 sensor enables this system to accurately gather environmental temperature and humidity data, thus establishing a solid foundation for intelligent and personalized temperature and humidity control.

The microcontroller unit (MCU) system is tasked with acquiring ADC signals and reading sensor data through the I^2^C protocol. To meet these requirements, the system requires a high-precision ADC channel and a general-purpose input/output (GPIO) interface capable of supporting I^2^C communication. To balance cost-effectiveness and performance, we selected the STM32F103C8T6 microcontroller unit, which is based on the ARM Cortex-M3 core. This microcontroller unit has a maximum clock frequency of 72 MHz and features direct memory access (DMA) functionality, significantly improving data transmission efficiency. Additionally, the MCU has two I^2^C interfaces to support multi-sensor data reading.^27^^,^^28^^,^^29^ Another key factor in choosing the STM32F103C8T6 is its powerful analog signal processing capabilities. The chip is equipped with two 18-channel 12-bit ADCs, enabling rapid sampling and accurate data transmission of sensor output analog signals, meeting the real-time monitoring and control requirements.^30^ Considering its cost-effectiveness, extensive peripheral resources, and good development support, the STM32F103C8T6 is an ideal choice for this system.

**Figure 2:**
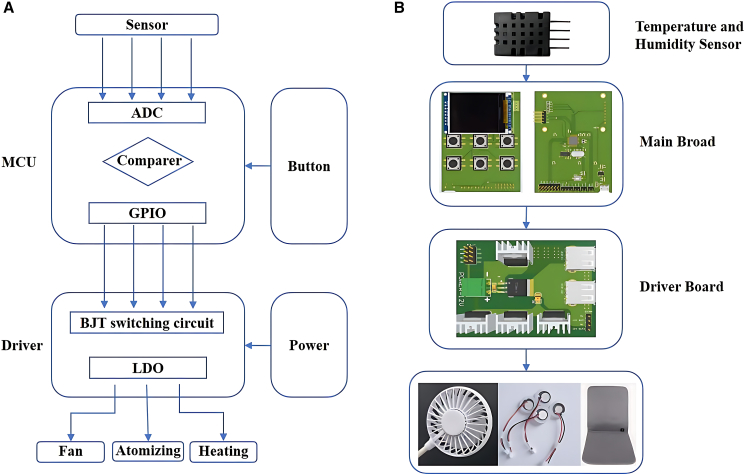
Design and implementation of an intelligent seat control system (A) System block diagram of the intelligent seat control. (B) Hardware components and layout of the intelligent seat system.

Figure 2 illustrates the working flow of the system’s sensors and actuators. The MCU board is powered by the AMS1117 module, which converts the external 5 V voltage to the 3.3 V voltage required by the MCU. The AMS1117 is a general-purpose low dropout regulator (LDO) that performs excellently in scenarios where the output current does not exceed 300 mA, with a standby current of only 2 μA and an output voltage error controlled within ±2%. Furthermore, it has a wide operating temperature range, functioning properly from −40°C to 125°C. To improve data acquisition accuracy, capacitors are configured at the input and output ends of the AMS1117 to filter out power supply ripple, enhancing the system’s stability.

The display is a 1.8-inch organic light-emitting diode (OLED) screen, which is driven using the SPI protocol.^31^ In SPI communication, the MCU connects to the display via four lines: serial clock (SCK), master-out slave-in (MOSI), master-in slave-out (MISO), and chip select (CS). The SCK line synchronizes the data transmission, with the MCU sending one bit of data per SCK clock cycle. The OLED screen samples or outputs data at the rising or falling edge of SCK to ensure synchronization. The MCU transmits commands and data to the OLED screen’s driver integrated circuit via the MOSI line, facilitating the display of information on the screen.

The humidifier is controlled through a pulse width modulation (PWM) signal generated by the drive circuit board, causing the piezoelectric ceramic to produce high-frequency oscillations.^32^ The generated PWM signal is transmitted to the piezoelectric ceramic through the drive circuit board, which usually contains a power amplifier or a dedicated driver chip to ensure that sufficient current and voltage can be provided to drive the piezoelectric ceramic plate. After receiving PWM signal, piezoelectric ceramic generates high frequency vibration through inverse piezoelectric effect. A piezoelectric ceramic plate vibrating at high frequencies breaks up the liquid touching its surface into tiny droplets. These droplets, usually within a few micrometers in diameter, form a fine mist of water. Such small droplets can be suspended in the air for a long time to achieve the effect of humidification.

The power management section of the drive board uses the LM1084 low dropout regulator to convert the input 12 V power to the 5 V required by the modules. The LM1084 has a maximum output current of 5 A and a linear adjustment rate of 0.015%, meeting the power management needs of the drive board.

Since the GPIO ports of the microcontroller cannot provide sufficient voltage to directly heat the seat cushion, drive the fans, and power the humidifiers, external drive chips are introduced into the system. Specifically, the system uses multiple TIP122 Darlington transistors to drive these high-current devices. TIP122 has a high current gain and a large collector current, making it ideal for loads that require large currents in drive systems. In addition, TIP122 is very sensitive to current changes and can respond quickly when the system status changes, ensuring that equipment such as heating cushions, fans, and atomizers can adjust their operating status in time. By selecting suitable power management and driving components, the system can effectively cope with the demand of high current load and ensure the normal operation of each function module.

#### Software algorithm design

To read data from the AHT20 sensor, the system first initializes the I^2^C bus. It then sends a measurement command (0xAC), reads the resulting six bytes of raw temperature (*S*_*T*_) and humidity (*S*_*Rh*_) data, and terminates the communication.

Subsequently, based on the AHT20 data format and conversion formulas,^33^ the system converts the raw data into actual temperature and humidity values. The temperature (*T*) conversion formula is as follows:

(Equation 1) $$T=\frac{S_{T}}{2^{20}}\times 200-50$$

The RH conversion formula is:

(Equation 2) $$\text{RH}=\frac{S_{Rh}}{2^{20}}\times 100\times 100\%$$

Through this procedural framework, the system accurately acquires and processes temperature-humidity microenvironment data from the AHT20 sensor attached to athletes’ skin surfaces. This ensures measurement precision and reliability, establishing a robust foundation for subsequent physiological status analysis and environmental regulation.

Push-button control is implemented using an interrupt-driven approach. A GPIO pin connected to the button is configured as an input, triggering an interrupt service routine (ISR) upon a press event. Within the ISR, the signal is first debounced via a software delay to prevent noise. Once the button press is confirmed, the system executes the corresponding user-defined action, such as switching display modes or adjusting temperature and humidity setpoints to meet the athlete’s personalized comfort demands.

Beyond manual device control, the adaptive intelligent regulation seat incorporates an automatic control mode. In this mode, sensors continuously collect microenvironmental data from athletes’ skin surfaces, which are processed by the microcontroller’s embedded control algorithm. The algorithm dynamically compares real-time temperature-humidity values against predefined physiological comfort thresholds. When either parameter exceeds the designated comfort zone, the system rapidly identifies anomalies and activates corresponding actuators for regulation, achieving precise environmental control. Previous studies indicate that optimal indoor resting conditions for athletes involve temperatures around 20°C with RH between 40% and 65%.^34^ Based on these findings, we established the following adaptive thresholds for automatic mode: low/high temperature thresholds (*T*_low_ = 19°C, *T*_high_ = 23°C) and RH boundaries (*H*_low_ = 40%, *H*_high_ = 65%).

## Results

### Intelligent seating system implementation

**Figure 3:**
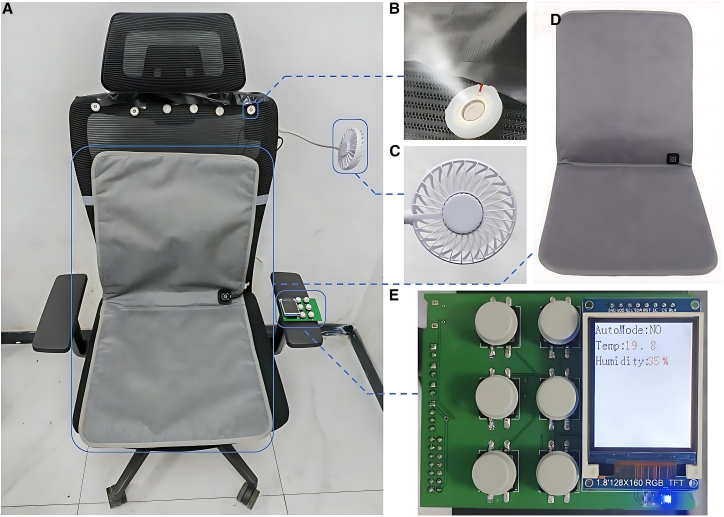
Intelligent seat system: component layout and function visualization (A) Intelligent seat system. (B) Humidifiers. (C) Fans. (D) Heating cushions. (E) LCD screen output results test.

The physical implementation of the intelligent seating system is illustrated in Figures 3A–3D. To validate system functionality, power-on testing was conducted using a 12 V DC power supply. The system demonstrated precise environmental monitoring capabilities by accurately measuring temperature and humidity parameters. As presented in Figure 3E, the LCD interface showed a RH reading of 35% and a temperature value of 19.8°C. For quantitative verification of data acquisition accuracy, comparative measurements were performed simultaneously using an independent high-precision hygrometer. The measurement results exhibited strong correlation with system readings (see Video S1), thereby preliminarily confirming both sensor reliability and display accuracy.

Video S1. Qualitative video demonstration of the intelligent adaptive seating system This video showcases the system’s operational workflow, user interface, and real-time responsiveness.

### Functional analysis of temperature and humidity capabilities for the system

**Figure 4:**
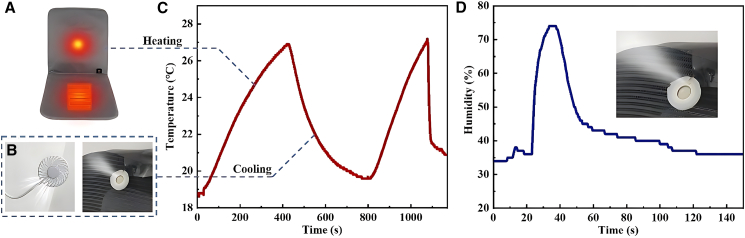
Functional analysis of the test system’s heating, cooling, and humidification capabilities (A) Seat heating system. (B) Seat cooling and humidification system. (C) Heating and cooling function results of the test system. (D) Verification of humidification function of the test system.

Further, we tested the heating and cooling functions of the system. The system’s heating is achieved through the heating pad, while cooling is realized by a fan. We first turn on the seat heating function, so that it starts to heat, and record the ambient temperature change read by the system. Then, we conducted the cooling function test. After the heating pad stopped working, we started the fan to lower the ambient temperature, continuing to record the temperature changes. Throughout the test, we recorded the ambient temperature data in detail, as shown in Figures 4A–4C. These data indicate that the heating pad can effectively increase the ambient temperature, while the fan can effectively lower it, thereby verifying the system’s temperature control function.

Additionally, we tested the humidification function. By triggering the key corresponding to the humidification function of the single-chip microcomputer system, the output signal drives the oscillation of the humidification plate through the driving circuit, generating atomized water vapor to increase the ambient humidity. The test results of the ambient humidity changes are shown in Figure 4D. We can see that after activating the humidification function, the ambient humidity rapidly increased by 38% within 11 s, achieving a good humidification effect. Meanwhile, during the process of reducing humidity, we turned off the humidification plate and started the fan, achieving a rapid decrease in the ambient humidity. These results demonstrate that the system can effectively control the ambient humidity in practical applications.

### Temperature and humidity adaptive function assessment of the system

**Figure 5:**
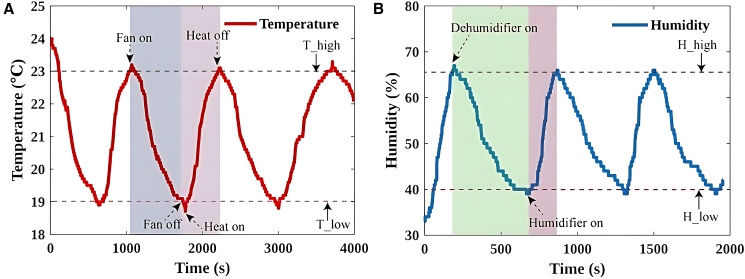
Adaptive function assessment of temperature and humidity regulation (A) Temperature adaptive function test. (B) Humidity adaptive function test.

Next, we tested the temperature and humidity adaptive functions. Figure 5A presents the dynamic response characteristics of the temperature adaptive control system of this intelligent seat system. The initial experimental condition was an ambient temperature of 24.0°C, which was higher than the preset high-temperature threshold *T*_high_ = 23.0°C. The system immediately triggered the cooling actuator, the fan, and the humidification module, and the temperature began to decrease slowly. When it dropped to the low-temperature threshold *T*_low_ = 19.0°C, the system switched to the heating mode, and the heating pad started, causing the temperature to rise back to around 23.0°C, forming a periodic oscillation behavior with an amplitude Δ*T* = 4.0°C. This oscillation pattern repeated three times within the experimental period, verifying the stability of the system within the set temperature range. Similar to the previously described temperature control mechanism, the humidity control system also adopted an immediate monitoring and dynamic adjustment method to achieve precise control of the ambient humidity.^35^ Figure 5B shows the dynamic characteristics of the humidity control system. In the initial stage, the ambient RH was at 35%, which was lower than the lower limit *H*_low_ of the system’s preset ideal humidity range. The humidification device was triggered to start. As the humidity rose and reached 66%, which exceeded the upper limit *H*_high_ of the ideal range, the humidity regulation device was activated to maintain the ambient humidity within the appropriate range. When the humidity dropped to *H*_low_, which was 40%, the low humidity threshold was triggered again, and humidification began. During the observation period, the humidity fluctuation pattern repeated three times, but each time it was precisely controlled between 40% and 65% according to the recommended optimal RH, which proved the reliability and effectiveness of the system.^36^^,^^37^ Therefore, the system achieves adaptive control of temperature and humidity through real-time monitoring and dynamic adjustment. This regulatory mechanism fully demonstrates the dynamic equilibrium capability of this adaptive control system in complex environments.

### Experimental verification of post-exercise skin thermo-hygrometric regulation by the system

To scientifically and systematically evaluate the practical efficacy of the intelligent seating system in regulating post-exercise cutaneous temperature and humidity, this study meticulously designed a three-phase experimental protocol centered on human body surface physiological indicators (temperature and humidity). The experimental workflow comprised preliminary preparation, exercise simulation, and data acquisition phases.

During the preliminary phase, the AHT20 temperature and humidity sensor array, the core device for physiological data acquisition, underwent rigorous calibration to ensure its measurement accuracy. Baseline physiological parameters including skin temperature, RH, and heart rate were recorded from subjects in a resting, relaxed state prior to formal experimentation. All trials were conducted under strictly controlled environmental conditions (25°C ambient temperature, 40% RH) to minimize external interference and enable accurate assessment of the seating system’s physiological intervention capabilities.

Subjects were required to perform 30-min moderate-intensity treadmill exercise wearing lightweight breathable athletic apparel to induce significant thermoregulatory responses (target core temperature elevation ≥1.5°C). Immediately post-exercise, participants transitioned to the intelligent seating system where calibrated AHT20 sensors were securely affixed to facial skin surfaces. Upon activation of the system, the embedded control algorithm immediately initiated the operation of the fan. At the same time, it meticulously and continuously recorded the cutaneous thermal and hygrometric data over a precisely defined 12-min acquisition period. This period was specifically designed to capture the critical transition phase from the hyperthermic peak to physiological stabilization. All acquired biometric data were stored in real-time for subsequent analysis. Concurrent with physiological monitoring, subjective thermal comfort was assessed using a standard 7-point thermal sensation vote (TSV) scale ranging from −3 “cold” to +3 “hot” at 1-min intervals.

**Figure 6:**
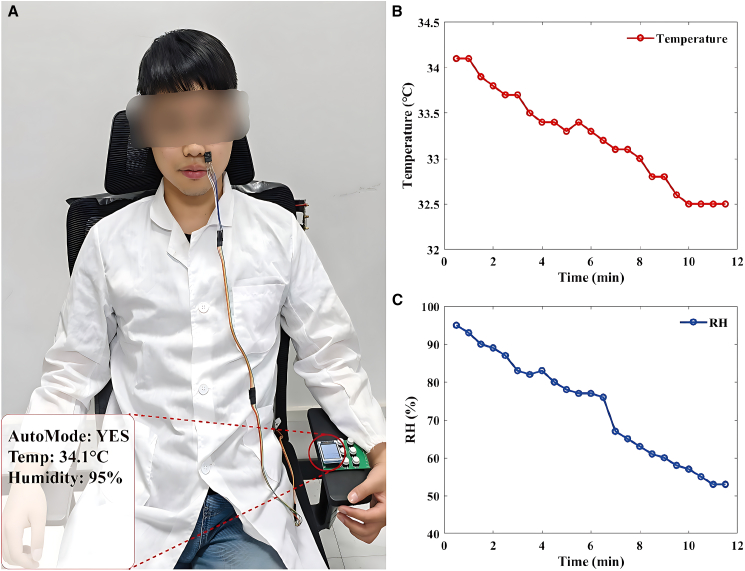
Dynamic changes in body surface temperature and RH post-exercise (A) Temperature and humidity measurements after exercise. (B) Curve of body surface temperature changes over time. (C) Curve of body surface RH changes over time.

Experimental validation of the seating system’s intelligent monitoring and regulation capabilities is presented in Figure 6. Following treadmill exercise, initial cutaneous measurements demonstrated significantly elevated physiological states compared to baseline: facial skin temperature reached 34.1°C with 95% RH,^38^^,^^39^ reflecting typical post-exercise hyperthermic-hyperhidrotic conditions. The active cooling mechanism commenced immediately upon system activation, inducing rapid reductions in both skin temperature and humidity as shown in Figures 6B and 6C. The most pronounced decline occurred during the initial minutes, followed by gradual stabilization. Quantitative analysis revealed that after 10 min of active regulation, average skin temperature decreased to 32.5°C while RH dropped to 53%. By the 12-min endpoint, both parameters exhibited continued downward trends toward equilibrium states. Notably, consistent with these objective measurements, subjective feedback quantified via TSV indicated that participants perceived a rapid transition from “hot “to “neutral” within the first 5 min. Compared with the typical passive waiting condition, this system demonstrates a significantly accelerated recovery process. In the passive waiting condition, natural physiological cooling mainly relies on environmental convection, and it usually takes a long time to reverse the state of excessive body temperature. These results indicate that this system can effectively alleviate post-exercise thermal-hygrometric state, suggesting a strong potential to promote physiological recovery through precise microclimate control.

## Discussion

This study has designed and implemented an adaptive, multifunctional intelligent regulation seating system to optimize athletes’ physiological status during pre-competition waiting periods, utilizing embedded technology and principles of physiological monitoring. The system incorporated an AHT20 temperature-humidity sensor, enabling accurate acquisition and reliable transmission of skin microenvironmental parameters, specifically temperature and humidity. The system monitors both ambient and the athlete’s personal microenvironment. It achieves rapid, precise regulation of temperature and humidity through intelligent control algorithms. The control logic maintains the microclimate within a range that promotes cooling while avoiding discomfort, aligning with the comfort zones defined in ASHRAE Standard 55, but adjusted for the higher metabolic heat output of post-exercise athletes. The system supports user-customized parameter adjustments through button interfaces. Additionally, its automatic mode intelligently manages module states based on real-time physiological data.

This intelligent, personalized, physiology-driven design aims to enhance perceptual comfort and recovery efficiency for athletes. Compared to traditional passive waiting, where recovery is often slow and uncomfortable, our active regulation approach provides a distinct advantage in stabilizing physiological states. While the current prototype is tailored for disciplines requiring rapid recovery and precise physiological control before competition, such as ice dance and figure skating, the underlying technology holds potential for broader application scenarios. These include rehabilitation settings where precise thermal management aids tissue recovery, as well as general athlete recovery protocols across various high intensity sports. It offers a novel technical approach and application paradigm for personalized physiological health management in smart sports venues.

However, it is equally crucial to define the scope of this study. This work aims to serve as a foundational study, with its main objective being to establish the technical feasibility of the proposed intelligent system and provide functional verification. Its core contribution lies in the successful design and implementation of the “proof-of-concept”: that is, constructing a closed-loop system capable of sensing real-time physiological microenvironment data and dynamically driving environmental regulation based on preset logic. Based on this successful verification, our next key stage will involve a comprehensive comparative study. Such studies are crucial for confirming the potential benefits proposed in this research through statistical data and will be the subject of our subsequent report.

### Limitations of the study

The experiments were conducted in a relatively controllable laboratory environment (ambient temperature 25°C, RH 40%). In contrast, the intended application scenarios described in the manuscript such as ice rinks involve specific and often harsh conditions, including low temperatures combined with high humidity or dry air. Consequently, it is unclear whether the system can deliver effective heating in cold environments or achieve sufficient dehumidification under extreme humidity, as these real-world conditions are not reflected in the current dataset. Additionally, the study assessed only facial skin temperature and humidity, which limits the physiological scope. Thermoregulatory recovery depends not only on peripheral responses but also on core body temperature restoration. Without direct core temperature monitoring, a decline in facial skin temperature cannot reliably indicate dissipation of internal heat, the skin may feel cool while core temperature remains elevated.

## Resource availability

### Lead contact

Requests for further information and resources should be directed to and will be fulfilled by the lead contact, Chong-ran Zhao (zhaocr@syty.edu.cn).

### Materials availability

This study did not generate new unique reagents.

### Data and code availability

The data supporting the findings of this study are available within the manuscript and the supplemental information. This study does not generate original code. Any additional information required is available upon reasonable request to the lead contact.

## Acknowledgments

The authors thank all subjects that participated in this series of experiments. This work was funded by the Open Project of the Key Laboratory of Textile Fiber and Products (10.13039/100018901Ministry of Education), 10.13039/501100012140Wuhan Textile University, no. Fzxw2024018.

## Author contributions

Conceptualization, C.-r.Z. and L.C.; methodology, C.-r.Z. and C.-r.L.; software, C.-r.L.; validation, L.Z., F.-s.W., and C.-r.L.; formal analysis, C.-r.Z., L.Z., and F.-s.W.; investigation, C.-r.Z., L.C., and L.Z.; resources, C.-r.Z., L.C., and C.-r.L.; data curation, C.-r.Z. and F.-s.W.; writing – original draft preparation, C.-r.Z. and L.C.; writing – review and editing, C.-r.Z., L.C., and C.-r.L.; visualization, C.-r.Z. and F.-s.W.; supervision, C.-r.Z.; project administration, C.-r.Z., L.C., and C.-r.L.; funding acquisition, C.-r.Z. All authors have read and agreed to the published version of the manuscript.

## Declaration of interests

The authors declare that they have no known competing financial interests or personal relationships that could have appeared to influence the work reported in this paper.

## STAR★Methods

### Key resources table

**Table:** 

REAGENT or RESOURCE	SOURCE	IDENTIFIER
**Software and algorithms**		

MATLAB R2024a	MatnWorks	https://www.mathworks.com/
Microsoft Excel	Microsoft	https://www.microsoft.com

**Other**		

AHT20 Temperature and Humidity Sensor	SZLCSC	C2757850
STM32F103C8T6 Microcontroller Unit	SZLCSC	C8734
AMS1117 Low Dropout Regulator	SZLCSC	C6186
TIP122 Darlington Transistor	SZLCSC	C16283
1.8-inch OLED screen	SZLCSC	C359940
Original code	This paper	N/A

### Experimental model and study participant details

The study was conducted in two phases: a preliminary pilot phase and a proof-of-concept validation phase. For the pilot study, five ice athletes (*n*=5, 3 males and 2 females) were recruited from Shenyang Sport University to determine optimal physiological comfort thresholds. For the proof-of-concept system validation, one healthy male volunteer (Age: 25 years; Height: 170 cm; Weight: 68 kg) was recruited to evaluate the system’s closed-loop dynamics. All participants were of East Asian ancestry and were free from cardiovascular, pulmonary, or thermoregulatory disorders. Participants were required to maintain a regular sleep schedule (>7 hours) and abstain from caffeine and alcohol for 24 hours prior to the experiments. During the experimental period, their routine physical training was suspended to ensure a stable baseline. All participants were fully informed of the experimental procedures and provided written informed consent. This study was approved by the Biomedical Ethics Committee of Hangzhou Dianzi University.

### Method details

#### System design and hardware

The study designed and implemented an adaptive, multifunctional intelligent control seat system. The system integrates several components: a temperature and humidity sensors (AHT20), a microcontroller unit, button control modules, and a display.

The system uses an STM32F103C8T6 microcontroller, which is based on the ARM Cortex-M3 core. It was chosen for its balance of cost and performance, and its capabilities for analog signal processing. The MCU has a clock frequency of 72 MHz and features DMA for efficient data transmission. An AHT20 temperature and humidity sensor is used to collect environmental data. It transmits data to the MCU via the I^2^C protocol. The sensor has a relative humidity accuracy of ±2% and a temperature accuracy of ±0.3°C at 25°C.The seat's built-in actuators for climate control include a heating pad, a fan, and a humidifier. These are driven by external drive chips, specifically TIP122 Darlington transistors, which are used to handle the high-current loads. A screen displays real-time data and the system's operating mode, driven by the SPI protocol. Buttons allow for manual control and parameter adjustments.

#### Software and algorithm design

The microcontroller's embedded adaptive control algorithm continuously compares real-time temperature and humidity readings against predefined physiological comfort thresholds. Simplified control logic pseudocode and core implementation files are provided in the supplemental information to ensure reproducibility.

Optimal resting conditions were identified as temperatures around 20°C and relative humidity (RH) between 40% and 65%. Based on this, the study set adaptive thresholds for the automatic mode: a low-temperature threshold of 19°C, a high-temperature threshold of 23°C, a low humidity boundary of 40%, and a high humidity boundary of 65%.When a parameter exceeds its comfort zone, the system activates the corresponding actuator (heating, cooling, or humidity regulation) to correct the environment and maintain it within the ideal range.

#### Sensor calibration and application protocol

To ensure the accuracy, reliability, and consistency of the physiological data collected, a strict calibration and application protocol was established and followed for all experimental sessions.

Sensor Calibration: Prior to the human trials, the accuracy of each AHT20 temperature and humidity sensor was individually verified. The sensors were placed in a controlled environmental chamber alongside a high-precision, calibrated reference instrument (testo 608 Temperature Humidity Meter; accuracy: ±0.5°C, ±3% RH). Data were logged simultaneously from both the AHT20 sensors and the reference instrument for 60 minutes under stable conditions (approximately 22°C and 50% RH). All sensors used in the study demonstrated readings within the manufacturer's specified tolerance when compared to the reference device, confirming their suitability for the experiment.

Sensor Placement: For all participants, the sensor was placed on the skin surface of the cheek, centered over the zygomatic arch (cheekbone). This location was chosen for several reasons: (1) it provides a relatively flat and stable surface for consistent sensor contact; (2) it is highly vascularized and responsive to changes in thermoregulation; and (3) it is located near the respiratory zone, making it relevant for assessing the microenvironment related to respiratory comfort. The placement site was marked to ensure consistency across the different phases of the experiment for each participant.

Skin Attachment Method: Before the sensor was attached, the designated skin area on the participant's cheek was gently cleaned with an isopropyl alcohol wipe and allowed to air dry completely. This step removed surface oils and residues to ensure proper adhesion and consistent thermal contact. The sensor assembly was affixed to the skin using double-sided, biocompatible medical adhesive tape (3M 1522 Double Sided Medical Tape). The tape was applied to the perimeter of the enclosure, ensuring firm and continuous contact between the sensor and the skin surface without placing pressure directly on the sensing element. This method prevented motion-induced noise and ensured that the sensor was measuring the cutaneous microenvironment rather than ambient conditions.

#### Selection of physiological comfort thresholds

The control thresholds for the automatic mode (temperature: 19–23°C; relative humidity: 40–65%) were determined through a two-stage process. Firstly, we referenced established standards and literature on human thermal comfort, such as ASHRAE Standard 55, which defines optimal comfort zones for indoor environments. We also consulted sports science literature indicating that slightly cooler conditions can facilitate post-exercise recovery.

Secondly, to refine these general guidelines for our specific application, we conducted a preliminary pilot study (n=5) involving athletes in a post-exercise state. Participants were exposed to various microclimates created by the seat, and their subjective feedback on thermal sensation, comfort, and perceived recovery was collected. The results from this pilot phase consistently indicated that the 19–23°C and 40–65% RH range was perceived as most effective for promoting rapid thermal comfort without inducing shivering (at the lower bound) or a feeling of clamminess (at the upper humidity bound). This empirically derived range was therefore adopted for the main study.

#### Experimental procedures and validation

##### The study conducted tests in three phases

System Functionality Tests: The system's ability to monitor temperature and humidity was tested and validated against an independent, high-precision hygrometer, showing a strong correlation between the readings. The heating pad and fan functions were tested by recording ambient temperature changes. The heating pad effectively increased the temperature, while the fan effectively lowered it. The humidifier function was tested by activating the device and recording a rapid increase in ambient humidity (38% increase in 11 seconds).

Adaptive Control Tests: The automatic mode was tested for both temperature and humidity control. The temperature control test showed the system cycling between the T_low_ of 19°C and T_high_ of 23°C, demonstrating a periodic oscillation. The humidity control test showed the system maintaining humidity between 40% and 65% by activating the humidity regulation mechanisms as needed.

Proof-of-Concept System Validation with Human Subject: To validate the system's closed-loop control dynamics and cooling efficiency under realistic physiological loads, a representative case study was conducted involving a healthy volunteer (Male, age 25). This functional verification phase aimed to demonstrate the engineering performance of the regulation logic rather than derive population-level statistics. The participant performed 30 minutes of moderate-intensity treadmill exercise to induce a thermoregulatory response. Immediately post-exercise, with the participant seated in the system under controlled conditions (25°C, 40% RH), the active cooling mode was initiated. Cutaneous temperature and humidity were logged continuously at 1-second intervals for 12 minutes to track the recovery trajectory. Concurrent with objective monitoring, the participant provided subjective thermal sensation votes at 1-minute intervals using a standard 7-point scale to assess perceptual recovery. These results were subsequently analyzed against a descriptive baseline of passive waiting conditions to contextualize the system's accelerated recovery performance. Post-exercise, initial skin temperature was 34.1°C with 95% RH. After 10 minutes of active regulation, the average skin temperature dropped to 32.5°C and RH dropped to 53%, demonstrating the system's effectiveness in mitigating post-exercise thermal-hygrometric state. Raw sensor data corresponding to this validation are provided in Data S1.

### Quantification and statistical analysis

All physiological and environmental data were processed and analyzed using MATLAB 2024a (MathWorks, Natick, MA, USA). Raw digital signals from the AHT20 sensor were converted into temperature and relative humidity values using the manufacturer-specified transfer functions (Equations 1 and 2 in the main text). For sensor validation, Pearson’s correlation coefficient was calculated to assess the consistency between the AHT20 readings and a high-precision reference hygrometer.

Continuous physiological variables, including facial skin temperature and local relative humidity, were logged at 1-second intervals during the 12-minute post-exercise recovery period. For quantitative analysis, these data were segmented into 1-minute blocks to calculate average values and track the recovery trajectory. Subjective thermal comfort was quantified using a 7-point Thermal Sensation Vote scale (-3 to +3), recorded at 1-minute intervals.

## Supplemental information

Data S1. Raw sensor measurements corresponding to the experimental validation presented in Figure 6

Data S2. Core control algorithm implementation
